# Psychological readiness and expectancy-based evaluations in IoT-adoption smart music classrooms

**DOI:** 10.3389/fpsyg.2026.1783925

**Published:** 2026-09-14

**Authors:** Zhang Huawei, Loh Sau Cheong, Suzana Ariff Azizan, Hashem Salarzadeh Jenatabadi

**Affiliations:** 1 Department of Science and Technology Studies, Faculty of Science, Universiti Malaya, Kuala Lumpur, Malaysia; 2 Department of Music, Dongchang College, Liaocheng University, Liaocheng, Shandong Province, China; 3 Department of Educational Psychology and Counseling, Faculty of Education, Universiti Malaya, Kuala Lumpur, Malaysia; 4 Department of Econometrics and Business Statistics, School of Business, Monash University Malaysia, Subang Jaya, Selangor, Malaysia

**Keywords:** expectancy-based beliefs, IoT adoption, music education, smart classrooms, technology readiness

## Abstract

The integration of Internet of Things technologies into smart classroom environments has become increasingly prominent in higher education, including within music education programs. However, limited research has examined how music students engage with IoT-enabled smart classrooms from both behavioral acceptance and psychological readiness perspectives, particularly in the Chinese higher education context. Drawing on an integrated framework combining the Unified Theory of Acceptance and Use of Technology and the Technology Readiness Index, this cross-sectional study examined IoT adoption intention among music students enrolled in Chinese universities. Survey data were collected from 1,020 undergraduate and postgraduate music students across 12 universities with established smart classroom infrastructures. Established multi-item instruments were used to measure expectancy-based beliefs, contextual factors, technology readiness, and IoT adoption intention. Structural equation modeling and multi-group moderation analysis were employed to examine direct, indirect, and gender-based relationships within the proposed framework. The findings indicate that performance expectancy, effort expectancy, facilitating conditions, and technology readiness were significantly associated with IoT adoption intention, whereas social influence was not. Technology readiness showed the strongest association with IoT adoption and was also significantly related to both performance expectancy and effort expectancy. In addition, performance expectancy and effort expectancy functioned as parallel mediators linking psychological readiness with IoT adoption intention, with selected expectancy-based relationships varying across gender groups. By integrating perspectives on behavioral acceptance and psychological readiness, this study contributes to educational psychology and technology adoption research by offering a context-sensitive explanation of IoT adoption intention in performance-oriented learning environments, such as music education. The findings also highlight the importance of aligning smart classroom implementation with students' psychological readiness, usability expectations, and institutional support systems.

## 1 Introduction

Digital transformation has become a central feature of contemporary higher education, with Internet of Things technologies increasingly embedded within teaching and learning environments. IoT-enabled smart classrooms integrate interconnected devices, sensors, and digital platforms that support real-time interaction, data exchange, and adaptive learning resources (Çela et al., 2025). These developments are particularly relevant to music education, where learning activities often involve performance practice, immediate feedback, and collaborative engagement. IoT-supported tools, such as smart instruments, sensor-based performance-monitoring systems, and intelligent classroom infrastructures, enable music students to engage with learning content in dynamic and interactive ways (Yan, 2026). In the Chinese higher education context, national initiatives promoting educational digitalisation have encouraged universities to adopt smart classroom technologies, including within music and arts programs (Yan, 2026). Despite the growing availability of IoT-enabled learning environments, variation remains in how music students engage with and adopt these technologies.

Existing research on technology adoption in educational settings has drawn mainly on established acceptance models, particularly the Unified Theory of Acceptance and Use of Technology (UTAUT) and its extensions. These studies have offered valuable insights into students' perceptions of performance expectancy, effort expectancy, social influence, and facilitating conditions in relation to digital learning systems (El Alfy and Kehal, 2024; Xue et al., 2024). However, the literature remains uneven in several important respects. First, many studies have examined general learning platforms, online learning systems, or broad smart classroom initiatives (Kurni and Srinivasa, 2024; Nie et al., 2023; Noor et al., 2023; Xu, 2024), while discipline-specific learning environments, particularly music classrooms, have received less systematic attention. Second, existing research often emphasizes technological infrastructure, system design, or institutional readiness (Alfoudari et al., 2023; Alzaidi, 2022; Dimitriadou and Lanitis, 2023), but gives comparatively limited attention to learners' psychological readiness, including confidence, anxiety, trust, openness, discomfort, and insecurity toward technology-supported learning. This gap is especially important in music education because music learning differs from many conventional academic subjects. Music students engage in embodied practice, repeated performance refinement, real-time feedback, peer observation, and instructor-guided correction. Their interaction with IoT-enabled smart classrooms may therefore involve not only evaluations of usefulness and ease of use, but also psychological readiness to trust, manage, and emotionally engage with technology-mediated feedback systems. As a result, examining expectancy-based evaluations alone may provide only a partial understanding of students' intention to adopt IoT in smart music classrooms. A more integrated perspective is needed to clarify how behavioral acceptance factors derived from UTAUT and psychological readiness factors derived from TRI jointly relate to IoT adoption intention in music learning environments. Addressing this gap, the present study focuses on Chinese university music students and investigates IoT adoption intention through an integrated framework that combines expectancy-based beliefs, contextual support, and psychological readiness.

To address these limitations, an integrated theoretical perspective that combines behavioral acceptance and psychological readiness offers a more comprehensive approach to examining IoT adoption in smart classrooms. UTAUT offers a well-established framework for understanding behavioral acceptance through constructs such as performance expectancy, effort expectancy, social influence, and facilitating conditions. While these constructs capture important situational and contextual perceptions, they do not explicitly reflect stable psychological tendencies that shape how individuals approach new technologies. The Technology Readiness Index (TRI) complements this perspective by focusing on users' mental readiness to engage with technological systems, as reflected in dimensions such as optimism, innovativeness, discomfort, and insecurity (Parasuraman, 2000; Parasuraman and Colby, 2015). These psychological orientations are particularly relevant in music education settings, where students' engagement with technology may be closely linked to feelings of control, confidence, and trust in technology-mediated performance environments.

Building on this integrated perspective, the present study examines IoT adoption in smart classrooms among music students at Chinese universities. The study investigates how behavioral acceptance factors derived from the UTAUT, and psychological readiness factors derived from the TRI are associated with IoT adoption. In addition, the study considers gender as a contextual moderator of the association between facilitating conditions and IoT adoption, reflecting ongoing discussions about gender-related differences in access to support and in technology use experiences in educational contexts. By integrating these perspectives, the study aims to contribute to educational psychology and technology adoption research by offering a holistic explanation of IoT adoption that accounts for both situational acceptance and psychological readiness.

The overall aim of this study is to examine IoT adoption in smart classrooms among music students in China using an integrated framework that combines behavioral acceptance and psychological readiness perspectives. The proposed framework positions performance expectancy, effort expectancy, social influence, and facilitating conditions as behavioral predictors derived from the UTAUT, while technology readiness, represented by optimism, innovativeness, discomfort, and insecurity, is conceptualized as a psychological antecedent associated with adoption behavior. Gender is incorporated as a moderating variable to examine potential differences in how facilitating conditions relate to IoT adoption. This framework provides a structured basis for analyzing IoT adoption in smart music classrooms from a psychological perspective.

## 2 Theoretical framework and hypothesis development

This study draws on the UTAUT to examine IoT adoption in smart music classrooms. UTAUT is a comprehensive framework for understanding technology-related beliefs and contextual factors associated with individuals' engagement with digital systems. In educational settings, UTAUT has been widely applied to examine how learners evaluate and engage with technology-supported environments. In the context of music education, where learning processes involve practice routines, performance feedback, and peer interaction, UTAUT constructs offer a valuable lens for examining students' perceptions of IoT-enabled smart classrooms.

### 2.1 UTAUT and IoT adoption in smart music classrooms

Performance expectancy refers to the extent to which individuals believe that using a technology is associated with enhanced task accomplishment or learning-related benefits (Venkatesh et al., 2003). In smart classroom research within general education systems, performance expectancy is commonly understood as learners' belief that technology-supported environments are associated with enhanced learning processes and task performance (Ngendahayo et al., 2024). Prior studies on smart classrooms have shown that students' evaluations of digital learning environments often center on whether technological systems support academic engagement, learning efficiency, and meaningful interaction with course content (Alhasan et al., 2023; Kim et al., 2025; Shi et al., 2023). Within this broader educational context, performance expectancy is a key expectancy-based belief that guides students' evaluation of the relevance of smart classroom technologies to their learning activities.

In smart music classrooms, performance expectancy refers to music students' perceptions that IoT-enabled tools, such as sensor-based instruments, real-time feedback systems, and digital learning platforms, are aligned with music learning practices. For music students, these perceptions are closely connected to learning goals such as skill development, performance accuracy, and reflective practice, which are central to music education (Chen and Tang, 2025). As music learning relies on repeated practice, performance monitoring, and feedback, students' evaluations of whether IoT-supported smart classrooms align with these learning needs become a salient consideration in their engagement with such environments (Guo, 2024). Accordingly, the following hypothesis is proposed:

***H1:*** *Performance expectancy is associated with IoT adoption in smart music classrooms*.

Effort expectancy refers to the extent to which individuals perceive a technological system as easy to understand and manageable to use (Venkatesh et al., 2003). In smart classroom research in general educational contexts, effort expectancy has been widely examined as an expectancy-based belief reflecting students' evaluations of system usability, interaction clarity, and the cognitive demands associated with technology-supported learning environments (Hu, 2022). In these contexts, students' engagement with smart classroom technologies is often associated with the degree to which such systems align with their existing learning practices and digital competencies (Ashraf et al., 2023).

In smart music classrooms, effort expectancy reflects music students' perceptions of the accessibility and usability of IoT-enabled systems during learning and practice. Music students frequently engage with multiple technologies simultaneously, including musical instruments, audio-processing software, and digital learning platforms, which places particular emphasis on usability and the management of cognitive demands (Zhang, 2025). In such environments, students' evaluations of whether IoT-enabled smart classroom systems are easy to use and compatible with established music-learning practices become especially important. Prior research in music education has highlighted that perceptions of ease of use play a central role in shaping students' willingness to engage with technology-mediated learning systems (Zhang, 2025). Based on this reasoning, the following hypothesis is proposed:

***H2:*** *Effort expectancy is associated with IoT adoption in smart music classrooms*.

Social influence refers to the extent to which individuals perceive that essential others consider the use of a technology to be appropriate or desirable (Venkatesh et al., 2003). In smart classroom research across general education systems, social influence is commonly examined as a social and normative factor through which students interpret expectations regarding technology use communicated by instructors, peers, and educational institutions (Kwet and Prinsloo, 2020). Within these contexts, students' engagement with smart classroom technologies is often associated with shared norms, instructional expectations, and peer practices surrounding digital learning environments (Kim et al., 2025).

In smart music classrooms, social influence reflects music students' perceptions of expectations and norms communicated through ensemble practice, peer observation, and instructor guidance. Music education is inherently social, with learning activities frequently occurring in collaborative and performance-based settings (Turchet et al., 2025). As a result, music students may closely monitor whether IoT-enabled smart classrooms are regarded as accepted or valued within their programs. In such contexts, perceptions of social expectations surrounding the use of IoT-supported learning environments become a relevant consideration in students' engagement with smart classroom technologies (Abdullayevna, 2025). Accordingly, the following hypothesis is proposed:

***H3:*** *Social influence is associated with IoT adoption in smart music classrooms*.

Facilitating conditions refer to individuals' perceptions regarding the availability of resources, support, and organizational infrastructure that enable the use of a technological system (Venkatesh et al., 2003). Within smart classroom research in general educational contexts, facilitating conditions are commonly examined as contextual perceptions of access to technological resources, availability of technical assistance, and institutional arrangements that support technology-enhanced learning environments (Huang et al., 2019; Selim et al., 2020). These perceptions provide a contextual basis for students to evaluate whether adequate support exists to engage with smart classroom technologies.

When considered within smart music classrooms, facilitating conditions encompass access to IoT-enabled devices, availability of technical support, opportunities for training, and institutional policies that support the use of technology in music learning. Music students frequently rely on specialized equipment and technical guidance during practice and performance, which makes perceptions of support particularly salient in this context (Zheng, 2025). In such learning environments, students' evaluations of whether sufficient resources and support are available to sustain engagement with IoT-based systems are important contextual considerations in their adoption of smart classroom technologies (Li, 2023). Based on this perspective, the following hypothesis is proposed:

***H4:*** *Facilitating conditions is associated with IoT adoption in smart music classrooms*.

### 2.2 Technology Readiness Index (TRI)

Technology readiness reflects individuals' psychological preparedness to engage with new technologies and to integrate them into everyday activities. Rather than focusing on system characteristics, the TRI emphasizes stable psychological orientations that shape how individuals approach and interpret technology-enabled environments (Parasuraman, 2000). In educational settings, technology readiness captures students' underlying beliefs, feelings, and dispositions toward technological systems, which may coexist alongside expectancy-based evaluations examined in technology acceptance models (Negm, 2023).

The TRI comprises four dimensions. Optimism refers to a positive outlook toward technology and a belief that technological systems enhance efficiency, control, and learning opportunities. In educational contexts, optimism reflects students' confidence that technology-supported environments align with their learning aspirations (Nafia et al., 2023). Innovativeness refers to an individual's tendency to be receptive to new technologies and to view oneself as capable of experimenting with emerging systems. For students, innovativeness is characterized by openness to exploring unfamiliar digital tools and learning modalities.

In contrast, discomfort reflects a perceived lack of control or a sense of being overwhelmed when interacting with technology. In learning environments that involve complex systems, such as IoT-enabled smart classrooms, discomfort may arise from concerns about managing multiple technological components simultaneously (Nafia et al., 2023). Insecurity refers to distrust of technology and concerns regarding reliability, accuracy, or data-related issues. In educational settings, insecurity may be associated with apprehension about system stability, performance tracking, or dependence on digital platforms.

Together, these four dimensions form an overall TRI that represents students' psychological readiness to engage with technological systems. Optimism and innovativeness reflect enabling orientations, while discomfort and insecurity represent inhibiting orientations (Kaushik and Agrawal, 2021). When considered collectively, the index provides a balanced assessment of students' mental readiness to engage with IoT-enabled smart classrooms beyond situational expectations or institutional support.

For music students, technology readiness is particularly relevant due to the distinctive characteristics of music learning. Music education relies on embodied practice, performance evaluation, and iterative feedback, often within technologically mediated environments. Students' psychological comfort, openness, and trust in technology may therefore shape their perceptions and engagement with IoT-enabled smart classrooms (Lyu and Sokolova, 2023). Assessing technology readiness enables consideration of these psychological dimensions alongside behavioral acceptance factors, thereby offering a more comprehensive understanding of IoT adoption in music education contexts. Based on this reasoning, the following hypothesis is proposed:

***H5****: TRI is associated with IoT adoption in smart music classrooms*.

### 2.3 Integrated research model

The present study develops an integrated research model that combines UTAUT and TRI to examine IoT adoption intention in smart music classrooms. UTAUT offers a behavioral acceptance perspective by explaining how students evaluate a specific technology through expectancy-based beliefs and contextual conditions. In this study, performance expectancy, effort expectancy, social influence, and facilitating conditions are positioned as direct predictors of IoT adoption intention. This means that students' perceptions of usefulness, ease of use, social expectations, and available support are examined as proximal evaluations associated with their intention to engage with IoT-enabled smart classroom technologies.

TRI complements UTAUT by adding a psychological readiness perspective. Unlike UTAUT constructs, which focus on evaluations of a particular technology or learning environment, TRI reflects students' broader psychological orientation toward technology. It captures enabling dimensions such as optimism and innovativeness, as well as inhibiting dimensions such as discomfort and insecurity. In the proposed model, TRI is positioned as a psychological antecedent associated with IoT adoption intention. This positioning assumes that students who are more psychologically ready to engage with technology may be more prepared to evaluate IoT-enabled smart classrooms positively and to express stronger intention to use them.

The model also proposes that TRI is associated with performance expectancy and effort expectancy. This suggests that students' general psychological readiness toward technology may be reflected in their more specific evaluations of IoT-enabled smart classroom systems. For example, students with greater optimism and innovativeness may perceive IoT technologies as more useful and manageable, while discomfort and insecurity may be linked to more cautious evaluations of usefulness and ease of use. Therefore, performance expectancy and effort expectancy are not only direct predictors of IoT adoption intention but also function as parallel mediators between TRI and IoT adoption intention.

This mediating structure clarifies the conceptual logic of the integrated model. TRI represents a broader layer of psychological readiness, whereas performance expectancy and effort expectancy represent proximal expectancy-based evaluations. The model does not propose serial mediation. Instead, performance expectancy and effort expectancy are examined as two parallel pathways through which psychological readiness is associated with IoT adoption intention. Social influence and facilitating conditions are retained as direct UTAUT-based predictors because they represent social and contextual aspects of the learning environment.

Based on this integrated perspective, the following hypotheses are proposed:

***H6:*** *TRI is associated with performance expectancy in smart music classrooms*.

***H7:*** *TRI is associated with effort expectancy in smart music classrooms*.

***H8:*** *Performance expectancy mediates the association between TRI and IoT adoption intention in smart music classrooms*.

***H9:*** *Effort expectancy mediates the association between TRI and IoT adoption intention in smart music classrooms*.

### 2.4 Moderating role of gender

Gender has been widely examined as a contextual factor in technology adoption research within educational settings. Prior studies suggest that male and female students may differ in learning preferences, technology-related confidence, and patterns of support utilization when engaging with digital learning environments (Ademi and Loshkovska, 2025; Matobobo, 2026). These differences are often reflected in how students interpret expectancy-based beliefs and contextual support in technology adoption models. Within the UTAUT framework, gender has been theoretically positioned as a moderator of multiple structural relationships, including performance expectancy, effort expectancy, and social influence. More recent studies continue to support the relevance of gender as a moderating factor across multiple pathways in educational technology adoption, including AI and digital learning systems (Ong and Lai, 2006; Tamilmani et al., 2021).

In addition, emerging research highlights the role of psychological readiness in shaping students' engagement with advanced technologies in educational contexts. Technology readiness has been shown to be associated with students' openness to and acceptance of digital systems, including AI and smart learning platforms. Recent empirical findings also indicate that gender may moderate the associations between technology readiness, expectancy-based beliefs, and technology adoption behavior, suggesting that students may differ in how they evaluate both the psychological and contextual aspects of technology use (Adewale and Ndwandwe, 2026; Nouraldeen, 2023). These findings support the view that gender-related differences are not limited to a single construct but may extend across multiple evaluative pathways within integrated models.

Despite these developments, limited research has examined the moderating role of gender in IoT adoption within discipline-specific contexts such as music education, particularly in smart classroom environments. Music learning involves specialized equipment, iterative feedback, and close interaction with technological systems, which may shape how students evaluate both expectancy-based beliefs and psychological readiness. Therefore, it is theoretically appropriate to examine gender as a moderator across multiple structural relationships within the integrated model.

Accordingly, gender is included in the present study as a moderating variable across the associations between technology readiness, expectancy-based beliefs, contextual factors, and IoT adoption. The following hypothesis is proposed:

**H10:** *Gender moderates the association between TRI, performance expectancy, effort expectancy, facilitating conditions, social influence and IoT adoption in smart music classrooms*. Figure 1 shows the research framework.

**Figure 1:**
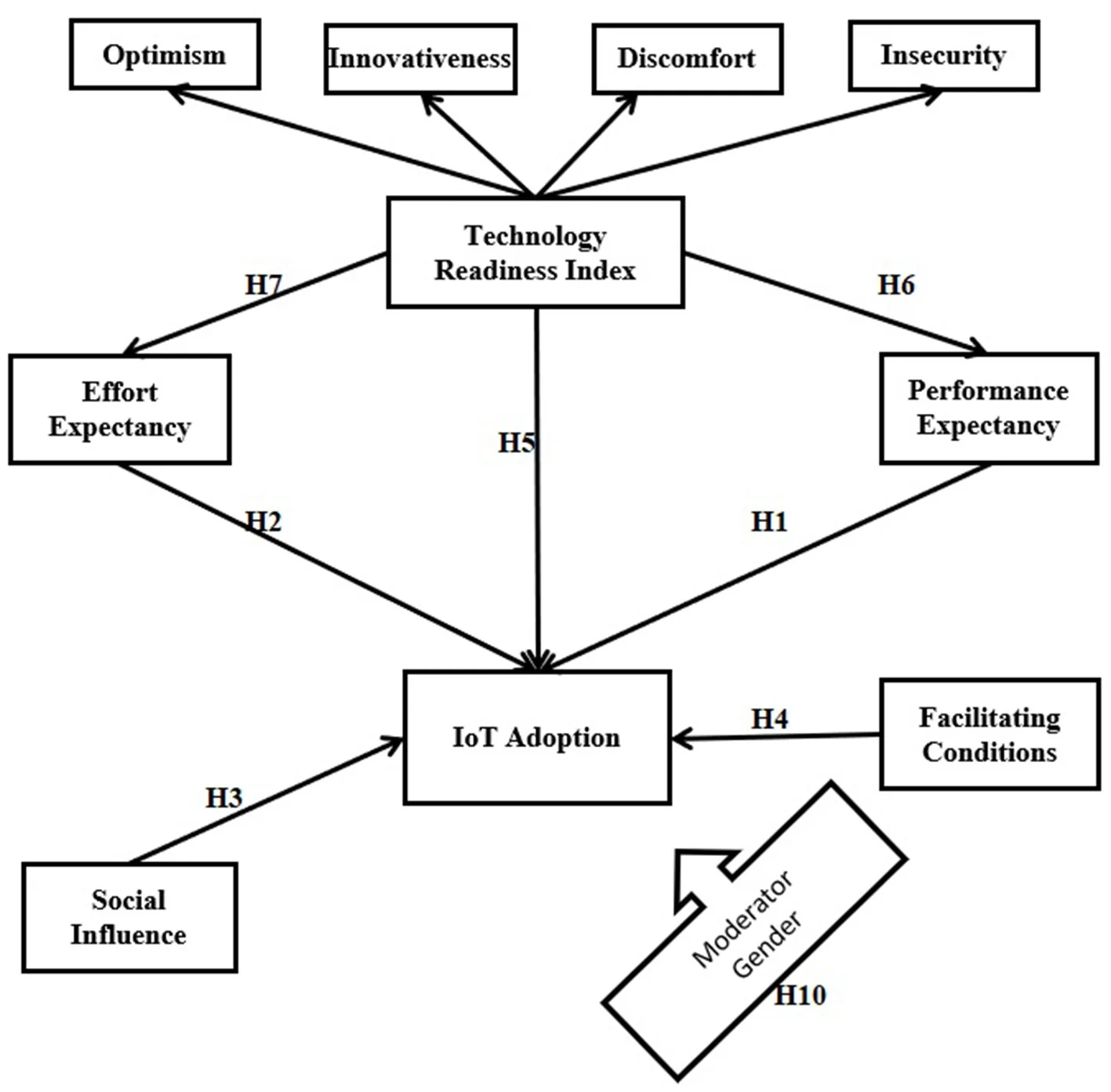
Research framework.

## 3 Method

This study employed a quantitative cross-sectional survey design to examine the relationships among psychological readiness, expectancy-based evaluations, contextual factors, and IoT adoption intention among music students in Chinese universities. This design was appropriate because the study aimed to test hypothesized relationships among latent constructs using SEM.

### 3.1 Ethical consideration

This study was conducted in accordance with established ethical standards for research involving human participants. Ethical approval was obtained from the University of Malaya Research Ethics Committee (Approval No. UM.TNC2/UMREC_3343). The review process confirmed that the study design, data collection procedures, and participant protections were consistent with institutional requirements and internationally recognized guidelines for research in educational and psychological contexts.

Prior to participation, all respondents were provided with clear and comprehensive information about the study's purpose, the voluntary nature of their involvement, and the procedures to be followed. Informed consent was obtained from all participants before data collection commenced. Participants were informed that they could decline to answer any question and could withdraw from the study at any time without penalty or negative consequences.

To protect participants' privacy and confidentiality, no personally identifiable information was collected at any stage of the study. All responses were fully anonymised at the point of data collection, and the dataset contained no information that could be used to identify individual participants. As a result, no additional consent for the publication of identifiable data was required. The collected data were used solely for academic research purposes, with access restricted to members of the research team. All data were securely stored in password-protected files in accordance with institutional data management and protection policies.

The study posed minimal risk to participants, as it focused on their perceptions and experiences of technology use in an educational setting. Participation was entirely voluntary, and informed consent was obtained from all respondents prior to data collection. No deception was involved, and no sensitive personal data was collected. The research procedures were designed to uphold participants' autonomy, dignity, and wellbeing throughout the study and to ensure full compliance with established ethical guidelines for research involving human participants.

### 3.2 Measure

Table 1 summarizes the measurement of the study constructs, the number of questionnaire items for each construct, and the sources in the literature. The constructs derived from the Unified Theory of Acceptance and Use of Technology were measured using established multi-item scales: performance expectancy (five items), effort expectancy (four items), social influence (four items), and facilitating conditions (four items), adapted from prior, validated studies. In this study, IoT adoption is operationalised as behavioral intention, which reflects students' stated likelihood of engaging with IoT-enabled smart classroom technologies. This approach is consistent with UTAUT-based research, where behavioral intention is commonly used as a proxy for adoption-related behavior. IoT adoption, operationalised as behavioral intention, was measured using three items drawn from the technology adoption literature.

**Table 1:** Measurement of constructs.

Construct	Number of questions	References
Performance expectancy	5	Al-Mamary et al., 2024; Momani, 2023; Raza et al., 2021
Effort expectancy	4	Raza et al., 2021; Al-Mamary et al., 2024; Momani, 2023
Social influence	4	Raza et al., 2021; Al-Mamary et al., 2024; Momani, 2023
Facilitating conditions	4	Raza et al., 2021; Al-Mamary et al., 2024; Momani, 2023
IoT adoption	3	Shaqrah and Almars, 2022
Optimistic	5	Alhasan et al., 2023
Innovativeness	4	Alhasan et al., 2023
Discomfort	4	Alhasan et al., 2023
Insecurity	4	Alhasan et al., 2023

In this study, the TRI was operationalised as a second-order latent construct comprising four first-order dimensions: optimism, innovativeness, discomfort, and insecurity. Consistent with prior research, optimism and innovativeness represent enabling dimensions, while discomfort and insecurity reflect inhibiting dimensions (Parasuraman, 2000). To ensure conceptual consistency, the inhibiting dimensions (discomfort and insecurity) were reverse-coded prior to analysis, so that higher TRI values indicate greater overall technology readiness. The second-order TRI construct was specified in the structural equation model using a reflective-reflective measurement approach, where the four dimensions serve as indicators of the higher-order construct. A higher-order confirmatory factor analysis was conducted to validate the second-order structure of TRI. All items were adapted from validated instruments and contextualized for smart music classroom settings. Responses were measured using a seven-point Likert scale ranging from 1 = strongly disagree to 7 = strongly agree.

All measurement items were adapted from well-established instruments to ensure content validity and consistency with prior research and were contextualized to reflect the use of IoT-enabled smart classrooms among music students.

### 3.3 Sample and sampling strategy

Participants were eligible if they were undergraduate or postgraduate music students enrolled in universities that had implemented smart classroom technologies in music education for at least 2 years. The study focused on students with prior exposure to smart classroom learning environments to ensure respondents could evaluate IoT-enabled technologies in relation to their learning experiences.

An adequate sample size is a critical requirement for structural equation modeling to ensure sufficient statistical power and stable parameter estimates. To determine the minimum sample size required for the proposed SEM model, an *a priori* power analysis was conducted using G^*^Power, a widely used software package in behavioral and social science research (Faul et al., 2007). The selection of effect size is a key consideration in power analysis, particularly for complex models with many predictors. While conventional guidelines categorize effect sizes of 0.10, 0.15, and 0.35 as small, medium, and large (Selya et al., 2012), respectively, the present study adopted a conservative effect size of 0.05. This decision reflects the model's complexity and reduces the likelihood of a Type II error.

The power analysis was performed assuming 37 predictors in the structural model, a significance level of 0.05, and a target statistical power of 0.80, which are commonly accepted thresholds in SEM-based research. Based on these parameters, the minimum required sample size was estimated to be 795 observations (see Figure 2). The final dataset comprised 1,020 music students, substantially exceeding the minimum requirement. This sample size is adequate to support reliable estimation of path coefficients, mediation effects, and moderation analyses within the SEM framework. Moreover, the achieved sample size enhances the precision and stability of model estimates, thereby strengthening the robustness of the empirical findings.

**Figure 2:**
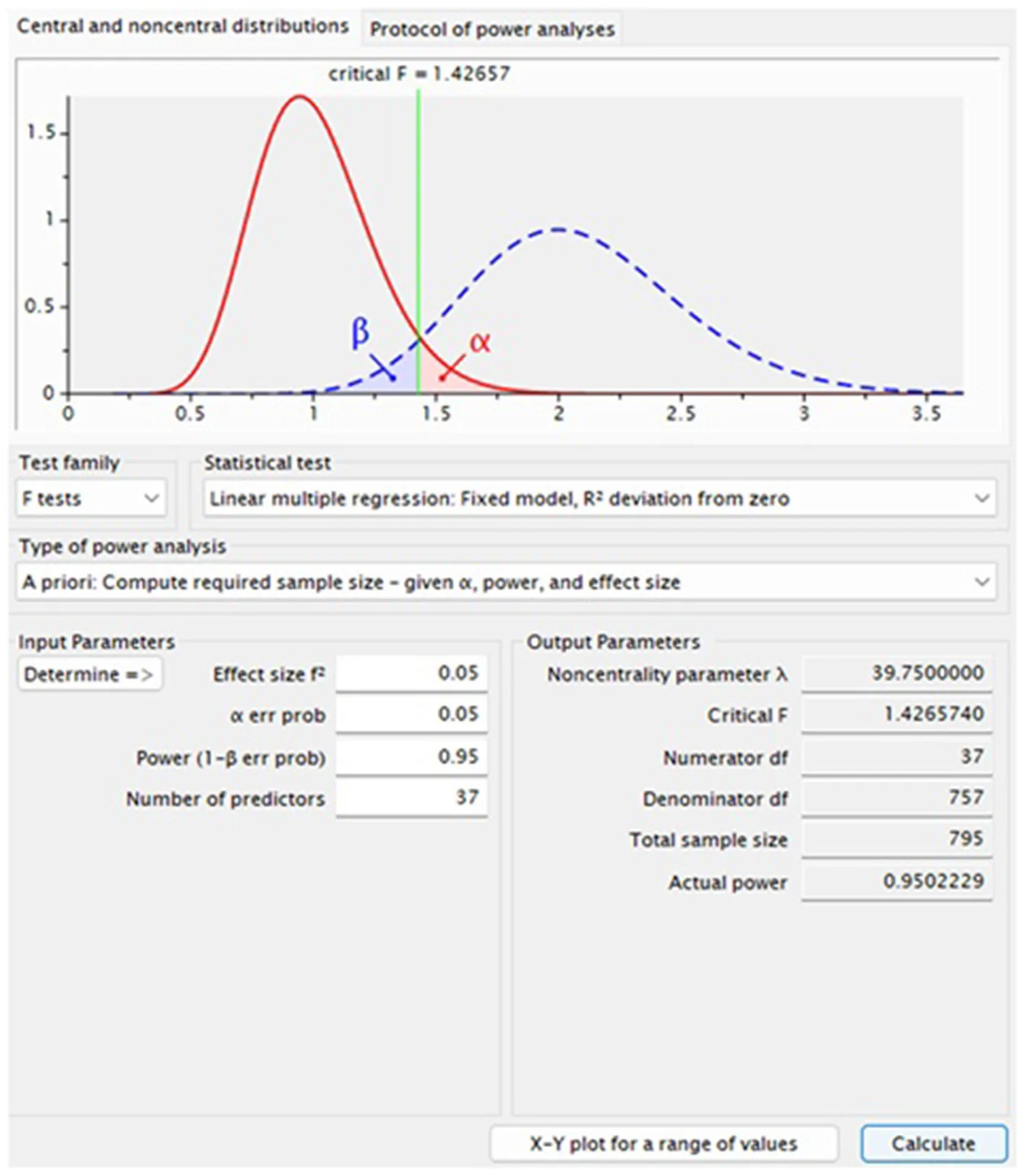
Sample size (G*Power).

### 3.4 Data collection procedure

The survey instrument was initially developed in English and subsequently reviewed for cultural and linguistic appropriateness by two bilingual individuals with high proficiency in both English and Chinese. To ensure translation accuracy and contextual equivalence, a rigorous forward-backwards translation procedure was employed. The original English version was first translated into Chinese, and an independent bilingual expert then translated the Chinese version back into English without reference to the original instrument. The two English versions were then carefully compared, and any discrepancies were discussed and resolved through consensus to preserve the original meaning and cultural relevance of the questionnaire items.

Following the completion of the translation, an online survey was developed to facilitate efficient, timely data collection. The survey link was distributed via WeChat and shared with undergraduate and postgraduate student groups across 12 Chinese universities that had implemented smart classroom technologies in music education for more than 2 years. This approach enabled access to a geographically diverse group of music students engaged in IoT-supported learning environments.

Given the recruitment approach used in this study, certain sampling limitations should be acknowledged. Participation was voluntary and relied on the dissemination of a WeChat-based survey, a widely used communication platform in Chinese higher education that may introduce self-selection bias. Students who elected to participate may differ from non-participants in motivation, digital engagement, or interest in music and in psychological research. Consequently, the final sample may not fully represent the broader population of music students across Chinese universities. Although online voluntary sampling is effective for reaching dispersed participants, future research may benefit from more structured sampling techniques or from using multiple recruitment channels to enhance representativeness.

Participation in the survey was entirely voluntary to ensure anonymity and encourage candid responses. This data collection strategy yielded 1,020 usable questionnaires, meeting the minimum sample size requirement. The achieved sample size provided sufficient statistical power to support the planned analyses and allowed for detailed examination of the study variables, thereby strengthening the overall rigor of the empirical investigation.

## 4 Results

### 4.1 Descriptive statistics

Table 2 presents the demographic characteristics of the study participants. The sample consisted of 1,020 music students, with female participants comprising 58% of the sample, compared with 42% male participants. The majority of respondents were undergraduate students, accounting for 78% of the sample, while postgraduate students comprised 22%. With respect to age, participants were distributed across multiple age groups, with 9.2% under 22 years old, 20.6% between 22 and 24 years old, and 28.5% over 27 years old. In terms of experience with smart classroom participation, 56% reported between 2 and 4 years, followed by 32% with more than 4 years and 12% with less than 2 years. Overall, the demographic distribution reflects a diverse sample across gender, academic level, age, and exposure to smart classroom environments in music education.

**Table 2:** Participant demographics.

Demographic	Number	Percentage
Gender	
Male	428	42%
Female	593	58%
Level of study	
Undergraduate	796	78%
Postgraduate	224	22%
Age	
Under 22 years old	367	36%
22–24	440	43%
Over 27 years old	213	21%
Experiences joining smart classroom	
Less than 2 years	121	12%
2–4 years	576	56%
More than 4 years	323	32%

### 4.2 Measurement model assessment (reliability, validity, and multicollinearity)

The measurement model was evaluated to assess reliability, convergent validity, discriminant validity, and multicollinearity, following established SEM guidelines (Hair et al., 2019).

Internal consistency was assessed using Cronbach's alpha and composite reliability (CR). As reported in Table 3, all constructs demonstrated acceptable reliability, with Cronbach's alpha and CR values exceeding the recommended threshold of 0.70. These results indicate satisfactory internal consistency across all measurement scales.

**Table 3:** Reliability and convergent validity.

Construct	Cronbach's alpha	Composite reliability	AVE
IoT adoption	0.752	0.823	0.534
Performance expectancy	0.786	0.851	0.586
Effort expectancy	0.772	0.839	0.571
Social influence	0.758	0.824	0.559
Facilitating conditions	0.833	0.887	0.631
Optimism	0.768	0.842	0.576
Innovativeness	0.781	0.854	0.547
Discomfort	0.794	0.861	0.603
Insecurity	0.739	0.821	0.522

Convergent validity was evaluated using factor loadings and average variance extracted (AVE). All item loadings were above 0.70, and AVE values exceeded 0.50 for all constructs, indicating that the latent variables adequately explain the variance of their respective indicators (see Table 3).

Discriminant validity was assessed using the Fornell–Larcker criterion. As shown in Table 4, the square root of AVE for each construct is greater than its correlations with other constructs, confirming that all constructs are empirically distinct. These results support the adequacy of discriminant validity in the measurement model.

**Table 4:** Discriminant validity (Fornell–Larcker criterion).

Construct	BI	PE	EE	SI	FC	TRI
IoT adoption intention (BI)	**0.731**					
Performance expectancy (PE)	0.58	**0.765**				
Effort expectancy (EE)	0.52	0.63	**0.756**			
Social influence (SI)	0.41	0.49	0.45	**0.748**		
Facilitating conditions (FC)	0.55	0.57	0.53	0.46	**0.794**	
Technology readiness (TRI)	0.69	0.66	0.71	0.48	0.59	**0.763**

The bold values on the diagonal represent the square root of the Average Variance Extracted (AVE) for each construct.

Multicollinearity was examined using the variance inflation factor (VIF). All VIF values were below the recommended threshold of 5, indicating that multicollinearity is not a concern in the model.

The TRI was modeled as a higher-order construct comprising four first-order dimensions: optimism, innovativeness, discomfort, and insecurity. Reliability and validity were assessed at both first-order and higher-order levels to ensure robustness of the measurement structure. For discriminant validity assessment, TRI was included at the higher-order level in the Fornell–Larcker matrix to maintain conceptual clarity.

### 4.3 Model fitting

To improve model fit, modification indices (MI) were examined, and only theoretically justifiable adjustments were applied. Specifically, a limited number of residual covariances were introduced between items within the same constructs, namely PE1 and PE2, and EE2 and EE3. These adjustments were supported by similarities in item wording and shared measurement context, which may lead to correlated measurement error. No cross-construct error covariances were specified. All measurement items were retained because they met acceptable loading thresholds and demonstrated theoretical relevance. These refinements were minimal and guided by theory to avoid data-driven overfitting.

Table 5 summarizes the model fit indices obtained before and after applying modification-index–based refinements. Before model refinement, the structural model demonstrated an overall acceptable level of fit, as most indices met commonly accepted thresholds in the SEM literature. Specifically, the initial values of CMIN/DF, GFI, TLI, and RMSEA indicated that the model provided a reasonable representation of the observed data. However, some indices, including CFI and PNFI, were closer to their lower acceptable bounds, suggesting that the model structure could be further optimized for comparative fit and parsimony.

**Table 5:** Model fitting analysis.

Indices	Value before MI	Value after MI	Terrible	Acceptable	Excellent	Evaluation
CMIN/DF	3.12	2.01	>5	>3	>1	Excellent
CFI	0.887	0.962	>0.8	>0.9	>0.95	Excellent
GFI	0.931	0.979	>0.8	>0.9	>0.95	Excellent
TLI	0.901	0.965	>0.8	>0.9	>0.95	Excellent
PNFI	0.587	0.721		>0.5		Acceptable
RMSEA	0.076	0.038		< 0.08	< 0.06	Excellent
SRMR	0.061	0.034	>0.1	< 0.08	< 0.05	Excellent

After examining the modification indices, we introduced theoretically justifiable adjustments to address localized misfit. These refinements included allowing selected error covariances between conceptually related indicators and removing redundancy associated with weaker measurement elements. As shown in the post-MI results, model fit improved consistently across all reported indices. CMIN/DF decreased from 3.12 to 2.01, indicating improved overall fit, while CFI, TLI, and GFI increased to values exceeding 0.95, reflecting stronger comparative and incremental fit. In addition, PNFI increased from 0.587 to 0.721, suggesting enhanced model parsimony. Notably, RMSEA decreased from 0.076 to 0.038, indicating a closer approximation to the population covariance structure. Collectively, these results support the adequacy of the refined model and provide a sound basis for subsequent structural analyses. The refined model also demonstrated an excellent SRMR of 0.034, below the recommended threshold of 0.05, indicating a good level of residual fit.

### 4.4 Structural model and hypothesis testing

Table 6 presents the results of the relationship analysis, reporting estimated standardized coefficients and corresponding 95% confidence intervals for the hypothesized direct and indirect associations in the proposed model. The direct effects indicate that performance expectancy is positively associated with IoT adoption (H1), as evidenced by a statistically significant coefficient and a confidence interval that excludes zero. Effort expectancy also shows a statistically significant association with IoT adoption (H2), although the magnitude of this association is relatively small. In contrast, social influence does not demonstrate a statistically significant association with IoT adoption (H3), as its confidence interval includes zero. Facilitating conditions are significantly positively associated with IoT adoption (H4), underscoring the importance of perceived institutional and technical support in smart classroom contexts.

**Table 6:** Direct effect.

Relationship	Estimated standardized coefficients	*P*-value
H1: Performance expectancy → IoT adoption	0.62^***^	< 0.001
H2: Effort expectancy → IoT adoption	0.16^*^	0.021
H3: Social influence → IoT adoption	0.09	0.168
H4: Facilitating conditions → IoT adoption	0.28^***^	< 0.001
Social influence → IoT adoption	0.09	< 0.001
H5: TRI → IoT adoption	0.83^***^	< 0.001
H6: TRI → Performance expectancy	0.63^***^	< 0.001
H7: TRI → Effort expectancy	0.79^***^	< 0.001

^*^p < 0.05; ^**^p < 0.01; ^***^p < 0.001.

In addition, technology readiness shows a strong, statistically significant association with IoT adoption (H5), indicating that psychological readiness toward technology is closely associated with students' engagement with IoT-enabled learning environments. Technology readiness is also significantly associated with both performance expectancy (H6) and effort expectancy (H7), suggesting that broader psychological orientations toward technology are reflected in expectancy-based evaluations of usefulness and usability.

The explanatory power of the structural model was assessed using R^2^ values for the endogenous constructs. The results indicate that the model explained 71% of the variance in IoT adoption intention, suggesting substantial explanatory power for students' intention to engage with IoT-enabled smart classroom technologies. In addition, technology readiness explained 40% of the variance in performance expectancy and 62% of the variance in effort expectancy, indicating that students' psychological readiness accounted for a meaningful proportion of their expectancy-based evaluations (see Table 7). These R^2^ values provide evidence that the integrated model has adequate explanatory strength and that both psychological readiness and expectancy-based beliefs are important components in understanding IoT adoption intention in smart music classrooms.

**Table 7:** Effect size.

Endogenous construct	*R^2^*
IoT Adoption intention	0.71
Performance expectancy	0.4
Effort expectancy	0.62

### 4.5 Mediation analysis

The mediation analysis examined whether performance expectancy and effort expectancy functioned as parallel mediators in the association between technology readiness and IoT adoption intention. Indirect effects were assessed using bootstrapped confidence intervals, with mediation considered statistically supported when the 95% confidence interval did not include zero. As shown in Table 8, the indirect pathway from TRI to IoT adoption intention through performance expectancy was statistically significant, indicating that students' psychological readiness toward technology was associated with IoT adoption, in part, through their evaluations of the usefulness of IoT-enabled smart classrooms. The indirect pathway through effort expectancy was also statistically significant, although weaker in magnitude, suggesting that perceived ease of use also contributed to explaining the association between technology readiness and IoT adoption intention.

**Table 8:** Indirect effect.

Relationship	Estimated standardized coefficients	*P*-value	LL 95% CI	UL 95% CI	Result
H8: TRI → Performance expectancy → IoT adoption	0.39^***^	< 0.001	0.26	0.53	Supported
H9: TRI → Effort expectancy → IoT adoption	0.13^*^	0.018	0.03	0.24	Supported

^*^p < 0.05; ^**^p < 0.01; ^***^p < 0.001.

The mediation pattern indicates partial mediation rather than full mediation. This is because the direct association between TRI and IoT adoption intention remained statistically significant after including performance expectancy and effort expectancy as mediators, while both indirect pathways were also significant. Therefore, technology readiness is associated with IoT adoption intention both directly and indirectly through students' expectancy-based evaluations of usefulness and usability. These findings support H8 and H9 and further confirm the value of integrating psychological readiness with expectancy-based evaluations in the proposed model (see Table 8).

### 4.6 Measurement invariance across gender

Prior to conducting gender-based comparisons, measurement invariance was assessed to ensure that the constructs were interpreted equivalently across male and female groups. Following established SEM procedures, configural and metric invariance were examined. Configural invariance was first evaluated by estimating the model separately for male and female groups without imposing equality constraints. The results indicated an acceptable fit for both groups (Male: χ^2^/df = 2.11, CFI = 0.953, TLI = 0.946, RMSEA = 0.041; Female: χ^2^/df = 2.08, CFI = 0.956, TLI = 0.949, RMSEA = 0.039), suggesting that the overall factor structure is consistent across groups. Metric invariance was then tested by constraining factor loadings to be equal across groups. The constrained model also demonstrated acceptable fit (χ^2^/df = 2.15, CFI = 0.951, TLI = 0.947, RMSEA = 0.042). The change in fit indices between the configural and metric models was minimal (ΔCFI = 0.002, ΔRMSEA = 0.001), remaining within recommended thresholds (ΔCFI < 0.01; ΔRMSEA < 0.015), indicating that metric invariance is supported. These results confirm that the measurement model operates equivalently across gender groups, supporting the validity of subsequent multi-group comparisons.

### 4.7 Moderation analysis

The moderation analysis examined whether structural relationships differed between male and female students using Wald tests, as reported in Table 9. The results indicate statistically significant gender-based differences for the associations between performance expectancy and IoT adoption and between effort expectancy and IoT adoption. Specifically, the Wald statistics for these two paths were significant, indicating that the strength and direction of these associations varied across gender groups. In contrast, the remaining paths, including those involving facilitating conditions, social influence, and technology readiness, did not show statistically supported differences between male and female students, as reflected by non-significant Wald test results. Overall, these findings suggest that gender moderates selected expectancy-based relationships with IoT adoption, while the associations related to technology readiness and contextual factors remain largely consistent across gender groups.

**Table 9:** Wald test results for moderation by gender.

Relationship	β Male	β female	SE male	SE female	Wald statistic	*p*-value	Moderation evidence
Performance expectancy → IoT adoption	0.55	−0.23	0.07	0.06	64.18	< 0.001	Yes
Effort expectancy → IoT adoption	0.07	−0.21	0.06	0.07	9.61	0.002	Yes
Facilitating conditions → IoT adoption	0.21	0.09	0.06	0.07	1.67	0.196	No
Social influence → IoT adoption	0.03	0.12	0.05	0.06	1.25	0.264	No
TRI → IoT adoption	0.89	0.85	0.08	0.07	0.15	0.697	No
TRI → Performance expectancy	0.58	0.49	0.06	0.06	1.13	0.288	No
TRI → Effort expectancy	0.76	0.67	0.07	0.07	0.83	0.362	No

## 5 Discussion

The present study set out to examine IoT adoption in smart music classrooms by integrating behavioral acceptance and psychological readiness perspectives within a single explanatory framework. Specifically, the study pursued three interrelated objectives. First, it explored how expectancy-based beliefs and contextual factors derived from the UTAUT are associated with IoT adoption among music students. Second, it investigated the role of technology readiness as a form of psychological readiness that accompanies students' engagement with IoT-enabled learning environments. Third, it examined the combined operation of these perspectives by assessing the mediating roles of expectancy beliefs and the moderating role of gender. Accordingly, the discussion is organized into three parts: an interpretation of UTAUT-based findings related to expectancy and contextual factors, an examination of technology readiness as a psychological foundation for adoption, and an integrated discussion of mediation and moderation patterns that together clarify how psychological readiness and expectancy-based evaluations coexist within smart music classroom contexts.

### 5.1 Expectancy-based beliefs and contextual factors in IoT adoption (discussion of UTAUT hypotheses H1–H4)

This subsection discusses the findings on expectancy-based beliefs and contextual factors derived from UTAUT, as specified in H1-H4. The results indicate that UTAUT remains useful for understanding music students' IoT adoption intention in smart classroom environments. The significant roles of performance expectancy, effort expectancy, and facilitating conditions are broadly consistent with prior UTAUT-based studies in educational technology and smart classroom contexts, where perceived usefulness, ease of use, and available support have been closely associated with students' engagement with digital learning systems (Alhasan et al., 2023; El Alfy and Kehal, 2024). These findings suggest that students' intention to adopt is related not only to the presence of smart classroom technologies but also to how students evaluate the learning value, usability, and institutional support associated with these technologies. At the same time, the present study extends prior work (Kim et al., 2025) by examining these relationships in smart music classrooms, a setting that remains underexplored, particularly within Chinese higher education. This context is important because music students' learning processes involve embodied practice, real-time feedback, repeated performance refinement, and interaction with specialized digital tools, which differ from more conventional classroom-based learning.

With respect to expectancy-based beliefs, performance expectancy and effort expectancy emerged as important evaluative dimensions associated with IoT adoption intention. This finding aligns with prior UTAUT research (Batucan et al., 2022), suggesting that students are more likely to intend to use educational technologies when they perceive them as useful and manageable for their learning tasks. In the music education context, performance expectancy may be particularly salient because IoT-enabled tools can be evaluated in relation to practice monitoring, performance awareness, feedback interpretation, and skill development. Similarly, effort expectancy is relevant because music students often interact with multiple technologies simultaneously, including musical instruments, audio-processing software, digital platforms, and smart classroom systems. Therefore, students' perceptions of whether these technologies are easy to use and compatible with established practice routines become central to their intention to adopt them. The gender-based differences observed in the group analyses further suggest that expectancy-based evaluations may not be interpreted uniformly across students. Rather than indicating that the technology itself functions differently, these variations may reflect differences in how students evaluate usefulness, usability, confidence, and learning support within IoT-enabled music learning environments.

Among the contextual factors, facilitating conditions were more clearly associated with IoT adoption intention than social influence. This pattern is meaningful when compared with broader e-learning literature, where social influence and facilitating conditions have both been examined as important UTAUT-based predictors, although the strength and consistency of social influence vary across educational contexts (Xue et al., 2024; Zheng et al., 2025). In contrast, the present findings suggest that music students' IoT adoption intention may be more closely aligned with direct evaluations of usefulness, usability, and available support than with peer or instructor expectations. One possible explanation is that smart music classrooms require hands-on interaction with specialized equipment, real-time feedback systems, and digital instruments. In such settings, students may place greater importance on whether the infrastructure works reliably, whether technical assistance is available, and whether the system supports their practice needs. Social influence may become less salient because students' judgments are shaped more by direct experience with the technology than by social pressure or normative expectations. Therefore, the non-significant role of social influence does not necessarily contradict UTAUT, but suggests that the relative importance of UTAUT constructs may vary across discipline-specific learning environments. Together, these findings indicate that expectancy-based beliefs and contextual support operate in complementary ways, with institutional conditions providing a practical foundation for IoT adoption intention in smart music classrooms.

### 5.2 Technology readiness as psychological readiness in smart music classrooms (discussion of TRI hypothesis H5)

This subsection addresses technology readiness as a form of psychological readiness associated with IoT adoption intention in smart music classrooms, as proposed in H5. Technology readiness reflects relatively stable orientations toward technology, including optimism, innovativeness, discomfort, and insecurity, rather than evaluations tied only to a specific system or learning task (Parasuraman, 2000). As such, technology readiness provides a foundational psychological context through which students approach technology-mediated learning environments (Negm, 2023). In this study, technology readiness is conceptualized as complementing UTAUT by capturing broader dispositions that shape how music students engage with IoT-enabled smart classrooms before and beyond situational expectancy-based evaluations.

The findings indicate that TRI exhibits the strongest association with IoT adoption intention in the general model and maintains stable associations across gender groups. This pattern suggests that psychological readiness may represent a more fundamental orientation toward technology engagement than individual expectancy-based beliefs alone. The strong role of technology readiness may be interpreted in terms of cognitive, motivational, and affective mechanisms. Cognitively, students with higher technology readiness may feel more capable of understanding and managing IoT-enabled systems, aligning with the demands of smart music classrooms, where learners interact with instruments, audio technologies, digital platforms, and real-time feedback systems. Motivationally, optimism and innovativeness may reflect openness to experimenting with new learning tools and confidence in technology-supported practice. These orientations may encourage students to view IoT-enabled classrooms as opportunities for improving practice monitoring, performance awareness, and reflective learning. Affectively, reduced discomfort and insecurity may lessen hesitation, uncertainty, or resistance to technology-mediated feedback, particularly when students are required to rely on digital systems during practice- and performance-related learning.

These mechanisms may be especially salient in music education because technology is not simply an external instructional aid but may become part of the learning and performance process itself. Music students often rely on repeated practice, self-monitoring, performance feedback, and emotional regulation when developing technical and expressive skills (Antonini Philippe et al., 2020). In this context, students' trust in technology, confidence in managing digital tools, and willingness to experiment with new systems may shape their responses to IoT-enabled smart classrooms. Conversely, concerns about system reliability, feedback accuracy, data interpretation, or dependence on digital platforms may reduce students' readiness to engage with such environments. Therefore, the stronger association between TRI and IoT adoption intention, compared with individual UTAUT indicators, may reflect the broader psychological role of readiness in shaping students' openness to technology-supported music learning.

The present findings extend existing research by addressing an underexplored area in technology adoption studies. Relatively few investigations have examined technology readiness within music education, smart classroom environments, or Chinese higher education contexts. By demonstrating the central role of technology readiness among music students, this study highlights the importance of incorporating psychological readiness when examining technology engagement in performance-based disciplines. Positioning technology readiness as a foundational construct contributes to a more comprehensive understanding of IoT adoption intention in smart music classrooms, particularly in learning contexts where emotional engagement, performance monitoring, and technology-supported self-regulation are prominent. This revised interpretation also strengthens the discussion by clarifying why TRI is not merely an additional predictor, but a psychological layer that helps explain how students approach and evaluate IoT-enabled learning environments.

### 5.3 Integrated role of technology readiness, expectancy beliefs, and gender (discussion of integrated hypotheses H6–H10)

This subsection examines the integrated role of technology readiness and expectancy-based beliefs in relation to IoT adoption, with attention to gender-based differences. Hypotheses H6 through H9 focused on the mediating roles of performance expectancy and effort expectancy in the relationship between technology readiness and IoT adoption. The proposed model specified parallel mediation, rather than a serial process, allowing each expectancy belief to function as an independent evaluative mechanism. Within this framework, technology readiness denotes a broad psychological orientation toward technology. In contrast, expectancy beliefs capture more proximal evaluations of anticipated learning value and perceived manageability in smart music classroom environments. This integration enables a clearer understanding of how general psychological readiness is reflected in context-specific evaluations without assuming a sequential or hierarchical ordering between mediators.

The mediation analysis revealed distinct patterns across gender groups. For male students, performance expectancy emerged as a more prominent mediator between technology readiness and IoT adoption, suggesting that evaluations of anticipated learning value and performance support play a central role in how psychological readiness is expressed. For female students, both performance expectancy and effort expectancy functioned as significant mediators, although the direction of these associations differed from those observed among males. These patterns can be interpreted as differences in evaluative processes rather than differences in the functionality or effectiveness of IoT-enabled systems. In music learning contexts, such differences may reflect variation in how students interpret the alignment between technology-supported environments and their individual learning routines, performance goals, and perceived demands.

The moderation analysis further indicated that gender selectively differentiated expectancy-based pathways, as shown in Table 7, while associations involving technology readiness remained stable across groups. This pattern suggests that gender-related differences are more pronounced in situational evaluations of usefulness and effort than in broader psychological orientations toward technology. The stability of technology readiness across gender groups reinforces its role as a foundational construct that underlies engagement with IoT-enabled smart classrooms. Differences in expectancy-based pathways may be linked to variation in learning styles, confidence in technology-mediated practice, and patterns of support utilization within music education. Together, these findings highlight the value of integrating technology readiness and expectancy-based beliefs to capture both stable psychological dispositions and context-sensitive evaluative processes, while recognizing that gender shapes how these evaluations are formed in smart music classroom settings.

## 6 Conclusion

This study advances technology adoption research by showing that IoT adoption intention in smart music classrooms is shaped not only by expectancy-based evaluations, such as perceived usefulness, ease of use, social expectations, and facilitating conditions, but also by students' broader psychological readiness toward technology. Situated within the context of Chinese higher education and music classroom digitalisation, the study integrated UTAUT and TRI to capture two complementary levels of explanation: students' situational evaluations of IoT-enabled smart classroom systems and their more stable psychological orientations toward technology.

The findings collectively suggest that expectancy-based beliefs and contextual factors remain relevant for understanding IoT adoption intention in smart music classrooms. Performance expectancy, effort expectancy, and facilitating conditions were associated with adoption intention, while technology readiness emerged as a particularly salient psychological foundation. The results also show that performance expectancy and effort expectancy function as evaluative pathways linking psychological readiness with adoption intention. In addition, gender-based analysis indicated that selected expectancy-based relationships varied across groups, whereas technology readiness showed more stable patterns. Together, these findings demonstrate the value of combining behavioral acceptance and psychological readiness perspectives to provide a more comprehensive and context-sensitive understanding of IoT adoption intention in performance-oriented learning environments such as music education.

### 6.1 Theoretical contributions

This study offers several theoretical contributions to educational technology adoption research by clarifying how expectancy-based evaluations and psychological readiness operate at different but complementary explanatory levels. UTAUT captures students' context-specific evaluations of IoT-enabled smart classrooms, including perceived usefulness, usability, social expectations, and available support. In contrast, TRI captures broader psychological orientations toward technology, including optimism, innovativeness, discomfort, and insecurity. By integrating these two perspectives, the study advances existing adoption models by showing that students' intention to adopt IoT in smart music classrooms is associated not only with immediate evaluations of the learning system, but also with their underlying readiness to engage with technology.

First, the study extends UTAUT into a discipline-specific setting by applying it to music education. Although UTAUT has been widely used in general educational technology and digital learning contexts, its application to performance-oriented learning environments remains limited. Smart music classrooms differ from conventional learning settings because students interact with musical instruments, digital platforms, real-time feedback systems, and performance-monitoring tools. By situating UTAUT within this context, the study demonstrates how performance expectancy, effort expectancy, social influence, and facilitating conditions operate in a music-learning environment where practice, feedback, and embodied performance are central.

Second, the study positions TRI as a psychological readiness layer that complements UTAUT rather than competing with it. The findings suggest that technology readiness provides a broader psychological foundation for understanding students' engagement with IoT-enabled learning environments. This contribution is important because UTAUT primarily explains adoption through situational beliefs about a specific technology, while TRI captures students' more stable orientations toward technology. In smart music classrooms, such readiness may be particularly relevant because students' engagement with IoT systems involves trust in feedback, confidence in managing digital tools, and openness to technology-supported practice.

Third, the study clarifies the dual role of performance expectancy and effort expectancy in the integrated model. These constructs operate not only as direct predictors of IoT adoption intention, consistent with UTAUT, but also as parallel mediators that link technology readiness to adoption intention. This finding adds theoretical depth by showing that psychological readiness may be reflected in students' evaluations of usefulness and ease of use. In this way, the model explains both the direct association between readiness and adoption intention and the indirect pathways through expectancy-based evaluations.

Finally, this study contributes empirical evidence from an underexplored context: Chinese smart music classrooms. Existing IoT adoption research has focused mainly on general student populations, broad smart classroom initiatives, or non-arts disciplines. By examining Chinese university music students, this study provides a more context-sensitive account of technology adoption in a performance-based educational setting. Collectively, these contributions strengthen the theoretical understanding of IoT adoption by integrating behavioral acceptance, psychological readiness, and discipline-specific learning conditions.

### 6.2 Practical implications

The findings of this study offer several practical implications for stakeholders involved in the design and implementation of IoT-enabled smart music classrooms. For music educators, the results suggest that IoT tools should be introduced gradually and connected clearly to students' existing music-learning routines. Rather than presenting smart classroom technologies as separate technical systems, educators can demonstrate how these tools support practice monitoring, feedback interpretation, performance improvement, and reflective learning. Guided demonstrations, short practice-based activities, and step-by-step explanations may help students understand how IoT-enabled systems can be used meaningfully during rehearsal, individual practice, and classroom-based performance tasks. This approach may be especially useful for students who are less confident or less psychologically ready to engage with new technologies.

For institutions, the findings highlight the importance of creating a supportive environment for IoT integration. Universities implementing smart music classrooms should provide regular technical support, orientation sessions, and hands-on training opportunities for both students and instructors. Institutional support should also include ensuring system reliability, maintaining equipment, and providing accessible assistance when technical problems occur. Because students may differ in their readiness to engage with smart classroom technologies, institutions should consider offering differentiated support, such as beginner-level training for students with lower confidence and more advanced workshops for students who are already comfortable experimenting with digital tools.

For smart classroom designers, the findings point to the need for systems that are intuitive, stable, and aligned with the cognitive demands of music learning. Interfaces should be simplified where possible, and real-time feedback should be presented in ways that students can easily interpret and apply to their practice. Designers should also consider how IoT systems interact with musical instruments, audio-processing tools, and digital platforms, so that students are not overloaded by multiple disconnected technologies. By reducing cognitive load, improving usability, and providing clear feedback, IoT-enabled smart classroom systems may better support students' psychological readiness and intention to adopt. Collectively, these implications emphasize that successful IoT implementation in music education requires not only technological infrastructure but also pedagogical alignment, institutional support, and learner-centered design.

### 6.3 Limitations and future research

Several limitations of this study should be acknowledged. First, although the study included music students from 12 Chinese universities, the voluntary WeChat-based recruitment strategy may limit the sample's representativeness. Students with stronger digital engagement, greater familiarity with smart classroom technologies, or higher interest in music-related technology research may have been more likely to participate. As a result, the findings should be interpreted with caution when generalizing to all music students in Chinese higher education or to students in other educational systems.

Second, the cross-sectional research design limits the ability to examine changes in technology readiness, expectancy-based evaluations, and IoT adoption intention over time. Because the data were collected at a single point in time, the study cannot determine how students' psychological readiness develops as they gain more experience with IoT-enabled smart classrooms. Future longitudinal research could examine whether optimism, innovativeness, discomfort, insecurity, performance expectancy, and effort expectancy change after extended exposure to smart classroom technologies.

Third, the use of self-reported survey data may introduce common method bias and social desirability bias. Although self-report measures are appropriate for examining students' perceptions and intentions, they may not fully reflect actual adoption behavior, frequency of use, or sustained engagement with IoT systems. Future studies could combine survey data with system-use records, classroom observations, or teacher evaluations to provide a more comprehensive understanding of IoT adoption in music education.

Future research may extend the present study in several directions. Longitudinal studies could clarify how psychological readiness and expectancy-based evaluations evolve across different stages of IoT implementation. Experimental or intervention-based studies could test whether guided demonstrations, technical training, peer support, or structured practice activities improve students' readiness and intention to adopt. In addition, qualitative or mixed-method research could provide deeper insight into how music students experience IoT-enabled feedback, digital performance monitoring, and technology-supported self-regulation. Cross-cultural and cross-disciplinary comparisons would also be valuable for examining whether the integrated UTAUT–TRI framework operates similarly in other educational settings, cultural contexts, or performance-based disciplines beyond music.

## Data Availability

The raw data supporting the conclusions of this article will be made available by the authors, without undue reservation.
